# Depression, Mental Distress, and Domestic Conflict among Louisiana Women Exposed to the *Deepwater Horizon* Oil Spill in the WaTCH Study

**DOI:** 10.1289/EHP167

**Published:** 2016-05-10

**Authors:** Ariane L. Rung, Symielle Gaston, Evrim Oral, William T. Robinson, Elizabeth Fontham, Daniel J. Harrington, Edward Trapido, Edward S. Peters

**Affiliations:** 1 Epidemiology Program,; 2 Biostatistics Program,; 3 Behavioral and Community Health Sciences Program, and; 4 Environmental and Occupational Health Sciences Program, Louisiana State University Health Sciences Center School of Public Health, New Orleans, Louisiana, USA

## Abstract

**Background:**

Psychological sequelae are among the most pronounced effects in populations following exposure to oil spills. Women in particular represent a vulnerable yet influential population but have remained relatively understudied with respect to the Deepwater Horizon oil spill (DHOS).

**Objective:**

To describe the relationship between oil spill exposure and mental health among women living in the southern coastal Louisiana parishes affected by the DHOS.

**Methods:**

The Women and Their Children’s Health Study administered telephone interviews to a population-based sample of 2,842 women between 2012 and 2014 following the DHOS. Participants were asked about depression, mental distress, domestic conflict, and exposure to the oil spill.

**Results:**

Over 28% of the sample reported symptoms of depression, 13% reported severe mental distress, 16% reported an increase in the number of fights with their partners, and 11% reported an increase in the intensity of partner fights. Both economic and physical exposure were significantly associated with depressive symptoms and domestic conflict, whereas only physical exposure was related to mental distress.

**Conclusions:**

This large, population-based study of women in southern coastal Louisiana, a particularly disaster-prone area of the country, revealed high rates of poor mental health outcomes. Reported exposure to the DHOS was a significant predictor of these outcomes, suggesting avenues for future disaster mitigation through the provision of mental health services.

**Citation:**

Rung AL, Gaston S, Oral E, Robinson WT, Fontham E, Harrington DJ, Trapido E, Peters ES. 2016. Depression, mental distress, and domestic conflict among Louisiana women exposed to the Deepwater Horizon Oil Spill in the WaTCH Study. Environ Health Perspect 124:1429–1435; http://dx.doi.org/10.1289/EHP167

## Introduction

An explosion on the *Deepwater Horizon* drilling rig on 20 April 2010 killed 11 people and caused almost 5 million barrels of oil to flow into the Gulf of Mexico. The spill covered 68,000 square miles of land and sea and triggered a response effort involving the use of nearly 2 million gallons of dispersant chemicals (U.S. Coast Guard 2011). Considered the largest accidental marine oil spill in history, the *Deepwater Horizon* oil spill (DHOS) resulted in widespread environmental and economic damage, the exact nature of which is only beginning to be understood.

In the wake of the DHOS, the Institute of Medicine called for research to generate evidence about the psychological and behavioral effects of oil spills (Institute of Medicine 2010). Previous disaster research has shown that psychological sequelae are among the most pronounced effects, with problems such as post-traumatic stress, depression, anxiety, and nonspecific distress figuring prominently in the literature (Acierno et al. 2007; Adams et al. 2006; Amstadter et al. 2009; Cerdá et al. 2013; DiGrande et al. 2011; Galea et al. 2007, 2008; Norris et al. 2002; Ruggiero et al. 2009). Disasters involving oil spills specifically have resulted in emotional consequences to people who live in the vicinity and rely on the affected areas for their economic and nutritional livelihoods (Lyons et al. 1999). For example, 1 year after the 1989 *Exxon Valdez* oil spill (EVOS), higher prevalences of generalized anxiety, post-traumatic stress disorder (PTSD), and depression were observed among the most highly exposed residents (Palinkas et al. 1993b). Individuals living in areas exposed to the 1996 *Sea Empress* oil spill were at higher risk of anxiety, depression, and worse mental health than individuals living in control areas (Lyons et al. 1999). Similarly, individuals exposed to the 2002 *Prestige* oil spill had an increased likelihood of reporting suboptimal scores in mental health (Carrasco et al. 2007; Sabucedo et al. 2010).

Early observations of psychological and economic harm immediately following the DHOS have also been reported (Buttke et al. 2012; Osofsky et al. 2011), with striking similarities in resultant health effects noted between the DHOS and previous spills (Gill et al. 2012). A 2011 survey conducted by the Gulf State Population Survey (GSPS) revealed that direct exposure to the oil spill itself was the most important determinant of mental health (Fan et al. 2015). Grattan et al. (2011) observed that greater spill-associated income loss was associated with greater depression, anxiety, and other mental health outcomes. Gill et al. (2012) reported that Alabama residents with greater exposure to the oil, greater economic loss, and commercial ties to natural resources also experienced higher levels of psychological stress. Reports of anxiety disorder, 14 or more mentally unhealthy days in the previous month, and stress about having enough money to pay for housing or food were also high (Gill et al. 2012). A report on behavioral health following the DHOS documented an increase in major depressive episodes, thoughts of suicide, and suicide plans from pre- to post-oil spill among persons 18–25 years old across the Gulf region [Substance Abuse and Mental Health Services Administration and Centers for Disease Control and Prevention (SAMSHA/CDC) 2013]. A study of female partners of oil spill cleanup workers revealed a higher prevalence of depression among those who had more physical contact with the oil and an increase in the number of domestic partner fights among those with both greater physical contact and economic exposure to the DHOS (Rung et al. 2015).

Experiences of domestic conflict and interpersonal violence among women have been associated with hurricane disasters (Anastario et al. 2009; Harville et al. 2011; Larrance et al. 2007) as well as with oil spills (Osofsky et al. 2010; Palinkas et al. 1993a). For example, the percentage of women reporting psychological victimization increased significantly from 34% before Hurricane Katrina to 45% after the storm, and hurricane-related stressors were a significant predictor of this increase (Schumacher et al. 2010). Psychological aggression is known to be a precursor to physical aggression in marriage (Cascardi et al. 1995; Murphy and O’Leary 1989), which in turn is linked to a variety of mental health disorders, including depression, post-traumatic stress disorder (PTSD), and suicidal ideation (Anastario et al. 2009; Coker et al. 2002; Forbes et al. 2014; Schumacher et al. 2010). Women are especially vulnerable to domestic violence, making the study of domestic conflict in the context of the DHOS and its related stressors particularly relevant.

These results all suggest substantial adverse mental health effects across various populations exposed to several different oil spills. Women in particular represent a vulnerable yet influential population. They are often central to decision-making processes within families, particularly with respect to decisions regarding health, support, diet, and child rearing, and women have remained relatively understudied with respect to the DHOS. [Men are the focus of the National Institute of Environmental Health Sciences (NIEHS)-funded Gulf Long-term Follow-up (GuLF) Study.] The objective of the present study was to describe the relationship between DHOS exposure and mental health among women living in the southern coastal Louisiana parishes that were affected by the *Deepwater Horizon* oil spill.

## Methods

### Study Design and Population

The Women and Their Children’s Health (WaTCH) Study is a longitudinal study of women in seven southern coastal parishes of Louisiana (Jefferson, Lafourche, Orleans, Plaquemines, St. Bernard, St. Mary, and Terrebonne) to assess the health effects of the DHOS. Data for the present analysis were from the first wave of interviews conducted between July 2012 and August 2014. Women were recruited randomly through an address-based sampling frame, with under-sampling from the larger, more urban parishes (Orleans and Jefferson). Volunteers were also accepted, although they comprised < 5% of the sample. Women were eligible to participate if they were between 18 and 80 years old, lived in the study area at the time of the oil spill, were the female head of household, had not participated in either the Louisiana Gulf Women’s Health Study (LGWHS) or the NIEHS GuLF Study (NIEHS 2012), and were mentally, physically, and linguistically able to complete the telephone interview. Potential participants were sent an introductory letter describing the study and inviting them to participate. Once enrolled, a 60-min computer-assisted telephone interview was administered, consisting of questions in medical, social, emotional, and behavioral domains. The response rate was 45%, as defined by the American Association for Public Opinion Research (AAPOR)(AAPOR 2011).

The WaTCH Study was reviewed and approved by the Louisiana State University Health Sciences Center (LSUHSC) internal review board and was granted a Waiver of Documentation of Informed Consent for the telephone interview. Study data were collected and managed using Research Electronic Data Capture (REDCap) electronic data capture tools hosted at the Epidemiology Data Center at the LSUHSC School of Public Health (Harris et al. 2009). REDCap is a secure, web-based application designed to support data capture for research studies, providing *a*) an intuitive interface for validated data entry; *b*) audit trails for tracking data manipulation and export procedures; *c*) automated export procedures for seamless data downloads to common statistical packages; and *d*) procedures for importing data from external sources.

### Measures

Exposure to the oil spill was measured using nine self-reported items (Table 1). Five items (Item 4 and Items 6–9) were adapted from prior work done on the *Exxon Valdez* oil spill (Palinkas et al. 1992) and subsequently used in other oil spill studies. The other four items were created for the present study. Because these nine items were highly correlated with each other, we used the data-driven approach of exploratory factor analysis to identify the factors (latent variables) that fit the variance–covariance matrix of the observed variables. For each indicator to be on the same scale, we dichotomized all nine questionnaire responses. Item 1 was based on the question “Did you or anyone in your household lose any income due to disruption of employment or closing a business because of the oil spill?” and was categorized based on yes/no responses. Item 2 was based on the question “Compared to other residents in your community, were you *1*) hit harder by the oil spill than others, *2*) affected about the same as others, or *3*) affected less than others?” Responses were grouped into “hit harder” versus “affected less or about the same.” Item 3 was based on the question “How would you rate the influence of the oil spill on your household’s current financial situation?” Responses were grouped into the following categories: “very or somewhat negative influence” versus “very or somewhat positive or no influence.” Item 4 and Items 6–9 were categorized based on yes/no responses. Item 5, which addressed smell, was created by combining questions on strength and frequency of smelling the oil and then was dichotomized based on “any smell” versus “no smell.” A two-factor solution had the best fit, explaining approximately 57% of the variance. The first factor, consisting of items related to income loss, how hard participants were hit compared with others, and the oil spill’s influence on household finances, was labeled “economic exposure.” The second factor, consisting of the other six items, was labeled “physical exposure.”

**Table 1:** Baseline characteristics of WaTCH Study sample, *n* = 2,852.

Characteristic	*n* (%)
Education
Less than high school	327 (11.8)
High school graduate	1,649 (59.3)
College or higher	804 (28.9)
Pre-oil spill household income
< 20,000 USD/year	645 (24.9)
20,000–50,000 USD/year	763 (29.5)
50,000–80,000 USD/year	545 (21.1)
> 80,000 USD/year	636 (24.6)
Race/ethnicity
Non-Hispanic white	1,522 (54.6)
Non-Hispanic black	945 (33.9)
Hispanic/multiracial/other	319 (11.5)
Marital status
Married/living with partner	1,785 (62.7)
Widowed/divorced/separated/never married	1,063 (37.3)
Currently employed
Yes	1,562 (59.1)
No	1,081 (40.9)
Louisiana Parish of residence at time of oil spill
Jefferson	489 (17.2)
Lafourche	540 (18.9)
Orleans	532 (18.7)
Plaquemines	184 (6.5)
St. Bernard	192 (6.7)
St. Mary	385 (13.5)
Terrebonne	530 (18.6)
Exposure to the DHOS
Economic exposure
1. Lost HH income as a result of employment disruption/closing of business because of oil spill	743 (26.2)
2. Hit harder by oil spill than others in community	167 (6.0)
3. Oil spill had somewhat or very negative influence on HH financial situation	1,064 (37.8)
Physical exposure
4. Oil spill caused damage to areas fished commercially	195 (6.8)
5. Extent and frequency of smelling oil
No smell exposure	1,694 (62.5)
Any smell exposure	1,016 (37.5)
6. Came into physical contact with oil in other ways (e.g., during home, recreation, hunting, fishing, or other activities)	624 (22.1)
7. Oil spill directly affected recreational hunting/fishing/other activities of household	972 (34.3)
8. Worked on any oil spill clean-up activities	55 (1.9)
9. Any property lost or damaged because of oil spill or cleanup	72 (2.5)
Age, years (mean ± SD)	45.7 ± 12.04
Time since DHOS, years (mean ± SD)	3.1 ± 0.38
Abbreviations: DHOS, *Deepwater Horizon* oil spill; HH, household; SD, standard deviation; WaTCH Study, Women and Their Children’s Health Study. Missing data: Education (*n *= 72); income (*n *= 263); race (*n *= 155); marital status (*n *= 4); current employment (*n *= 209); lost income (*n *= 15); hit harder (*n *= 56); negative influence (*n *= 40); smell (*n *= 142); physical contact (*n *= 28); lost/damaged property (*n *= 4).	

***Outcomes.*** Three mental health outcomes were assessed: depression, mental distress, and domestic conflict. Depression was measured using the 20-item Center for Epidemiological Studies Depression (CESD) Scale (Radloff 1977), with an established cutoff score of 16 suggestive of depressive symptoms (Eaton and Kessler 1981; Frerichs et al. 1981; Radloff 1977). Mental distress was measured using the Kessler-6 (K6) instrument (Kessler et al. 2002, 2003), with scores ≥ 13 indicating probable serious mental distress (Aldworth et al. 2005; Kessler et al. 2003) and scores between 8 and 12 indicating moderate mental distress (Galea et al. 2007; Kessler et al. 2003). Respondents were also asked if there had been an increase in the number of verbal or physical fights with their partner and if there had been an increase in the intensity of fights since the oil spill.

***Covariates.*** Age was assessed in years as a continuous variable. Household income was reported for the year immediately preceding the oil spill and was grouped into four categories: ≤ 20,000 USD/year, between 20,000 and 50,000 USD/year, between 50,000 and 80,000 USD/year, and > 80,000 USD/year. Race was grouped into three categories: non-Hispanic white, non-Hispanic African American, and Hispanic/multiracial/other (which also included Asian/Pacific Islanders and Native Americans). Education was measured as less than high school, high school graduate, and college or higher. Because the interviews occurred 2–4 years after the spill, we calculated the time between 20 April 2010 and the date of the interview and examined the effects of exposure while adjusting for this interval. The interval had no appreciable effect on depression or mental distress; therefore, it was excluded from those models and used only for the domestic conflict outcomes.

### Statistical Analysis

Statistical analyses were performed using SAS 9.4 (SAS Institute Inc.), except for the exploratory and confirmatory factor analyses, which were conducted using MPlus Version 7. Descriptive statistics were calculated for mental health outcomes, exposure items, and potential covariates. Poisson regression models with robust variances were used to calculate relative risks (RRs) and 95% confidence intervals (CIs) of the association between oil spill exposure and depression and domestic conflict. For mental distress, we used separate Poisson regression models with robust sandwich estimators of the variance to calculate RRs and 95% CIs to predict moderate and severe mental distress (vs. none) and severe mental distress (vs. moderate and none). All models were adjusted for age, race, income, and education. Time since spill was also adjusted for in the domestic conflict models and was not related to either depression or mental distress.

## Results

### Study Population

Table 1 presents the demographic characteristics of the participants. The mean age was 45.7 years [standard deviation (SD) 12.04]. The majority of women had graduated high school but not college (59%), were non-Hispanic white (55%), and were married or living with a partner (63%). Pre-oil spill income among the participants was relatively evenly distributed among the four income groups, and 59% of the women were currently employed. The distribution of participants by parish generally mirrored that in the 2010 census for women of similar age in the study area; for example, Plaquemines and St. Bernard parishes yielded proportionally fewer respondents, reflecting their smaller population sizes. Participants were interviewed on average 3.1 years (SD 0.38) after the oil spill.

### Exposure

Exposure to the DHOS was grouped into economic or physical exposure (Table 1). The major items contributing to economic exposure consisted of reports that the oil spill had a somewhat or very negative influence on household finances (38%) and reports of lost household income owing to a disruption of employment or to the closing of a business because of the oil spill (26%). The major items contributing to physical exposure consisted of reports that the spill had directly affected recreational activities (34%), smelling the oil (37%), and coming into physical contact with the oil in other ways (22%).

### Mental Health Outcomes

Table 2 describes the mental health outcomes of the study sample. The average CESD depression score was 11.8 (SD 12.46). When grouped using the standard cutoff of 16, > 28% of the women in the sample had depressive symptoms. The mean K6 score on mental distress was 6.1 (SD 5.30). Furthermore, > 13% of the sample scored in the severe mental distress range, and another 19% scored in the moderate mental distress range. With respect to domestic conflict, 16% of participants with a partner reported that the number of fights they had with their partners had increased since the oil spill, and 11% reported that the intensity of fights had increased.

**Table 2:** Mental health characteristics of WaTCH Study population, *n* = 2,852.

Characteristic	*n* (%)
Depression (CESD) score (mean ± SD)	11.8 ± 12.46
Depression groups
Presence of depressive symptoms (≥ 16)	766 (28.1)
No depressive symptoms (< 16)	1,964 (71.9)
Serious mental distress (K6) score (mean ± SD)	6.1 ± 5.30
Mental distress groups
Severe mental distress (≥ 13)	368 (13.2)
Moderate mental distress (5–13)	538 (19.4)
No mental distress (< 5)	1,874 (67.4)
Domestic conflict
Number of fights with partner has increased since oil spill^*a*^	369 (15.8)
Intensity of fights with partner has increased since oil spill^*b*^	265 (11.4)
Abbreviations: CESD, Center for Epidemiological Studies Depression scale; K6, Kessler-6 instrument; SD, standard deviaiton; WaTCH Study, Women and Their Children’s Health Study. Missing data: Depression (*n *= 122); mental distress (*n *= 72); number of fights with partner (*n *= 72), intensity of fights with partner (*n *= 76). ^***a***^Among those who reported having a partner, *n *= 2,330. ^***b***^Among those who reported having a partner, *n *= 2,326.	

### Predictors of Depression

The model showing the relationship between depression and exposure to the oil spill while adjusting for relevant covariates is shown in Table 3. Women reporting greater economic exposure to the oil spill were 1.2 (95% CI: 1.02, 1.41) times as likely to exhibit depressive symptoms as women who were not economically exposed, and women reporting greater physical exposure were 1.2 (95% CI: 1.01, 1.43) times as likely to exhibit depressive symptoms as women who were not physically exposed. Greater pre-oil spill household income and higher education level were protective for depressive symptoms. Hispanic, other race or multiracial women were 1.3 (95% CI: 1.1, 1.5) times as likely to exhibit depressive symptoms as white women.

**Table 3:** Adjusted associations with depression, *n* = 2,533.

Characteristic	Depressive symptoms (CESD ≥ 16) RR (95% CI)
Exposure to oil spill
Economic exposure	1.2 (1.02, 1.41)
Physical exposure	1.2 (1.01, 1.43)
Pre-oil spill household income (reference: ≤ 20,000 USD/year)
20,000–50,000 USD/year	0.7 (0.6, 0.8)
50,000–80,000 USD/year	0.5 (0.4, 0.7)
> 80,000 USD/year	0.4 (0.3, 0.5)
Race (reference: non-Hispanic white)
Non-Hispanic black	1.1 (1.0, 1.3)
Hispanic/multiracial/other	1.3 (1.1, 1.5)
Education (reference: less than high school)
High school graduate	0.7 (0.6, 0.8)
College or higher	0.6 (0.4, 0.7)
Age, years	0.99 (0.99, 1.00)
Abbreviations: CESD, Center for Epidemiological Studies Depression scale; CI, confidence interval; RR, relative risk.	

### Predictors of Mental Distress

The relationship between mental distress (severe vs. moderate/none and severe/moderate vs. none) and exposure to the oil spill while adjusting for relevant covariates is shown in Table 4. The results indicate that whereas the effect of physical exposure to the oil spill was significant, the effect of economic exposure was not. Women reporting more physical exposure to the oil spill were 1.4 (95% CI: 1.1, 1.8) times as likely to exhibit signs of severe mental distress (vs. moderate/none) and 1.2 (95% CI: 1.1, 1.4) times as likely to exhibit signs of severe or moderate mental distress (vs. none) as women reporting less physical exposure. Greater income and education were protective against mental distress, whereas race/ethnicity and age were not associated.

**Table 4:** Adjusted associations with mental distress, *n* = 2,571.

Characteristic	Mental distress (K6)^*a*^			Mental distress (K6)		
	Severe vs. moderate/none			Severe/moderate vs. none		
	RR	95% CI	95% CI	RR	95% CI	95% CI
Exposure to oil spill
Economic exposure	1.3	1.0	1.7	1.0	0.9	1.1
Physical exposure	1.4	1.1	1.8	1.2	1.1	1.4
Pre-oil spill household income (reference: ≤ 20,000 USD/year)
20,000–50,000 USD/year	0.6	0.4	0.7	0.8	0.7	0.8
50,000–80,000 USD/year	0.4	0.3	0.5	0.7	0.6	0.8
> 80,000 USD/year	0.3	0.2	0.5	0.6	0.5	0.7
Race (reference: non-Hispanic white)
Non-Hispanic black	0.8	0.6	1.0	1.0	0.9	1.1
Hispanic/multiracial/other	1.0	0.7	1.3	1.1	1.0	1.3
Education (reference: less than high school)
High school graduate	0.6	0.4	0.7	0.8	0.8	0.9
College or higher	0.3	0.2	0.4	0.8	0.7	0.9
Age, years	1.0	1.0	1.0	1.0	1.0	1.0
Abbreviations: K6, Kessler-6 instrument; CI, confidence interval; RR, relative risk. ^***a***^Serious mental distress defined by K6 scores ≥ 13; moderate mental distress defined by K6 scores between 8 and 12; no mental distress defined by K6 scores < 8.						

### Predictors of Domestic Conflict

The models showing the relationship between domestic conflict (increases in number and intensity of fights) and exposure to the oil spill while adjusting for relevant covariates are shown in Table 5. Both economic and physical exposure to the oil spill were associated with increased domestic conflict. Women reporting economic exposure to the spill were 1.7 (95% CI: 1.3, 2.1) times as likely to report an increase in the number of fights with their partners since the spill as women who were not economically exposed and were 1.6 (95% CI: 1.2, 2.2) times as likely to report an increase in the intensity of fights. Women reporting physical exposure to the oil spill were 1.7 (95% CI: 1.3, 2.2) times as likely to report an increase in the number of domestic fights and 1.6 (95% CI: 1.2, 2.2) times as likely to report an increase in the intensity of domestic fights as women who were not physically exposed. Higher income was protective against both types of domestic conflict, whereas more education was protective only against an increase in intensity. Women self-reporting as Hispanic, multi-racial, or other race were more likely to report an increase in the intensity of domestic fights than non-Hispanic white women.

**Table 5:** Adjusted associations with increased number and increased intensity of partner fights.

Characteristic	Increase in number of fights (*n* = 2,168) RR (95% CI)	Increase in intensity of fights (*n* = 2,166) RR (95% CI)
Exposure to oil spill
Economic exposure	1.7 (1.3, 2.1)	1.6 (1.2, 2.2)
Physical exposure	1.7 (1.3, 2.2)	1.6 (1.2, 2.2)
Time since spill, years	1.2 (0.9, 1.5)	1.4 (1.1, 1.9)
Age, years	1.0 (1.0, 1.0)	1.0 (1.0, 1.0)
Income (reference: ≤ 20,000 USD/year)
20,000–50,000 USD/year	0.8 (0.6, 1.0)	0.8 (0.6, 1.0)
50,000–80,000 USD/year	0.6 (0.5, 0.9)	0.6 (0.4, 0.9)
> 80,000 USD/year	0.7 (0.5, 0.9)	0.6 (0.4, 0.9)
Race (reference: non-Hispanic white)
Non-Hispanic black	1.0 (0.8, 1.3)	1.1 (0.8, 1.5)
Hispanic/multiracial/other	1.3 (1.0, 1.7)	1.6 (1.2, 2.2)
Education (reference: less than high school)
High school graduate	0.9 (0.7, 1.2)	0.9 (0.6, 1.2)
College or higher	0.9 (0.6, 1.2)	0.6 (0.4, 0.9)
Abbreviations: CI, confidence interval; RR, relative risk.		

## Discussion

This study, conducted 2–4 years after the *Deepwater Horizon* oil spill, revealed a high prevalence of poor mental health outcomes among women in southern coastal Louisiana. The prevalence of depressive symptoms as measured by the CESD was 28%, and the prevalence of severe mental distress as measured by the K6 was 13%. Domestic conflict, measured by an increase in the number of fights with a partner since the oil spill, was reported by 16% of respondents, and an increase in the intensity of partner fights was reported by nearly 11% of respondents.

### Depression

Increased rates of depression after disasters have been well-documented (Arata et al. 2000; Buttke et al. 2012; Carrasco et al. 2007; Cerdá et al. 2013; Galea et al. 2002; Lyons et al. 1999; McLeish and Del Ben 2008), and the results of the present study confirm these findings. Using the same metric, though not directly comparable, only ~20% of individuals in the general population would be expected to exhibit depressive symptoms (Radloff 1977). Estimates from the 1970s ranged from 21% of women in a nationally representative sample (Eaton and Kessler 1981) to 23.5% of women in a multi-ethnic probability sample in Los Angeles County (Frerichs et al. 1981); a 1997 study on premenopausal women in Boston demonstrated a prevalence of 22.4% (Harlow et al. 1999). Within a disaster context, many other studies have shown unusually high rates of depression/depressive symptoms compared with population norms (Arata et al. 2000; Buttke et al. 2012; Carrasco et al. 2007; Cerdá et al. 2013; Galea et al. 2002; Lyons et al. 1999; McLeish and Del Ben 2008). It is difficult to draw exact comparisons with the present study because of differences in sample selection, measurement instrument, and the nature of the trauma itself. However, the LGWHS, conducted in the same parishes as in the present study during approximately the same time period, found a prevalence of depressive symptoms of 31%; that study differed from the present one in that it enrolled women who were wives or partners of cleanup workers, a potentially more highly exposed sample (Rung et al. 2015).

Although it is difficult to compare rates across studies, depression has been consistently associated with exposure to oil spills (Arata et al. 2000; Carrasco et al. 2007; Lyons et al. 1999; Palinkas et al. 1992, 1993b; Sabucedo et al. 2010) and a variety of other disasters (Norris et al. 2002), including typhoons (Amstadter et al. 2009), hurricanes (Davis et al. 2010; McLeish and Del Ben 2008), earthquakes (Cerdá et al. 2013), and terrorist attacks (Galea et al. 2002), with a variety of ways used to quantify exposure. Our study adds to this body of work in finding that high depressive symptoms, after adjustment for characteristics commonly related to depression, were associated with greater physical and economic exposure to the DHOS. The results of other studies corroborate this finding. The LGWHS found a similar pattern of association between depression and physical exposure to the DHOS (Rung et al. 2015). Grattan et al. (2011) found that depression was associated with income loss caused by the DHOS. Similarly, Fan et al. (2015) showed a relationship between current depression and DHOS-related job and income loss, as well as a relationship between depression and direct contact with oil.

It is striking that depression was found to be so high in this population. It is possible that respondents already exhibited higher rates of depression before the DHOS than did their counterparts because of exposure to previous disasters such as Hurricanes Katrina and Rita. Indeed, national survey estimates from 2008 show that depression, measured by a different metric [the Patient Health Questionnaire (PHQ-8)] than the one used in the present study, was slightly higher in Louisiana compared with the national average (9.3% vs. 8.9%) (Reeves et al. 2011). Immediately following the oil spill in 2010–2011, Louisiana’s coastal parishes exhibited an even higher rate of depression: 16.4% on the PHQ-8 (SAMSHA/CDC 2013). A lack of pre-oil spill data precludes comparisons that could directly link depression to the oil spill in our sample. Nevertheless, our results are consistent with other evidence showing that populations residing closer to the epicenter of the DHOS and reporting more exposure are exhibiting higher-than-normal rates of depressive symptoms.

### Mental Distress

The present study found that the prevalence of severe mental distress (13%) as measured by the K6 was higher than expected. Nationally in 2009, ~4% scored in the severe range, and in 2007, the prevalence in Louisiana was 5.3% (Reeves et al. 2011). Rates of severe mental distress among adults in 32 counties in the Gulf Coast Disaster Area pre- and post-DHOS were 6% and 5%, respectively, showing little change (SAMSHA/CDC 2013). The LGWHS, in contrast, found a prevalence of severe mental distress of 12% (Rung et al. 2015), and a late-2010 survey of 4 coastal Louisiana parishes found a prevalence of 15% (Osofsky et al. 2011); both of these findings are quite similar to those of the present study. Rates begin to approach those found in the WaTCH Study as one moves closer in location and time to the DHOS.

Physical exposure to the oil spill was a predictor of mental distress in the WaTCH Study. This observation is contrary to findings of no association between any kind of exposure and mental distress among partners of oil spill workers in the LGWHS, although this lack of association may be a result of the smaller sample size in the LGWHS (Rung et al. 2015). The GSPS found no significant differences in severe mental distress from pre- to post-spill in 25 coastal counties of four affected states (SAMSHA/CDC 2013). These 25 counties, however, comprise all the counties bordering the Gulf Coast from Louisiana to the Florida panhandle, including the seven in the present study. Without an estimate of exposure, however, it is possible that the sample included many residents who were unexposed, thereby diluting any potential effect. Osofsky et al. (2011), in contrast, found positive associations between the disruption of participants’ lives by the DHOS and severe mental distress. Again, a lack of pre-oil spill data prevents us from attributing the rates found in the present study solely to the oil spill, but data from Hurricane Katrina documented increases in mental distress using the same instrument in the same areas before and after the storm (Kessler et al. 2006). Indeed, even higher rates of severe mental distress than those found in the present study were identified following Hurricane Katrina (Galea et al. 2007; Sastry and VanLandingham 2009), suggesting that the lingering effects of previous disasters may have contributed to making this population particularly vulnerable to a new disaster such as the DHOS.

### Domestic Conflict

The items for domestic conflict used in the present study are not comprehensive descriptors of partner violence. Nevertheless, the findings that 16% of respondents reported an increase in the number of fights with their partners and that 11% reported an increase in their intensity reflect an indication of marital/partner discord, which could escalate to more serious problems. Relationship conflict is known to be associated with male-to-female physical abuse (Schumacher et al. 2001) and intimate partner violence (IPV), which in turn is associated with a myriad of adverse mental health consequences (Coker et al. 2002), such as depressive symptoms (Cascardi et al. 1992; Cascardi and O’Leary 1992), depression (Cascardi et al. 1995; Gleason 1993), PTSD (Cascardi et al. 1995; Forbes et al. 2014; Gleason 1993; Kemp et al. 1991), and generalized anxiety disorder (Gleason 1993) as well as numerous somatic symptoms (Follingstad et al. 1991). Approximately 1.5 million women are raped and/or physically assaulted by an intimate partner in the United States each year (Tjaden and Thoennes 1998), making this a serious public health issue.

Significant life stressors, such as disasters, are known risk factors for partner aggression (Schumacher et al. 2001). Both economic and physical exposure to the DHOS were significantly associated with increased domestic conflict in the present study. These findings are consistent with data showing that disasters are related to intimate partner violence (Anastario et al. 2009; Harville et al. 2011; Larrance et al. 2007; Palinkas et al. 1993a; Palinkas 2012; Schumacher et al. 2010). Studies conducted after Hurricane Katrina of displaced persons living in FEMA trailers found a rate of IPV three times higher than U.S. baseline rates (Larrance et al. 2007); this rate increased a further 5% a year later (Anastario et al. 2009). Women experiencing IPV also had an increased risk of major depression and suicidal ideation (Anastario et al. 2009). Similarly, Harville et al. (2011) found that greater exposure to Hurricane Katrina was associated with an increased likelihood of violent methods of conflict resolution among postpartum women in Louisiana. Even within the context of an oil spill disaster, similar patterns are found with respect to domestic violence. Residents who were highly exposed to the EVOS were significantly more likely to report conflict with their spouses or partners and to report increases in domestic violence in their community (Palinkas et al. 1993a; Palinkas 2012). After the DHOS, qualitative interviews and focus groups with community stakeholders showed evidence of increased fighting and domestic violence (Osofsky et al. 2010). Likewise, among partners of DHOS cleanup workers, the prevalence of increased number of partner fights was 33%, which was associated with both physical and economic exposure to the oil spill (Rung et al. 2015). More research related to domestic conflict and intimate partner violence after the DHOS needs to be conducted because the conditions for further negative public health impact are present.

An alternative hypothesis for the present study’s results is that the relationship between oil spill exposure and the mental health outcomes is nonlinear. We explored this possibility by testing the effects of the squared exposure terms and by comparing model fit indices to models without the squared terms (data not shown). The quadratic models did not substantially improve the model fit, and the effect estimates did not substantially change with the squared terms. The exception was for severe or moderate mental distress, where physical exposure and the square of physical exposure were both significant, suggesting the possibility of a leveling out of the effect of exposure on distress at higher levels of distress. Because the sample size at the very high ends of the scales was limited, further exploration of this possibility is warranted in more appropriately powered studies.

Several limitations of this study should be noted. First, no data on prior history of mental health outcomes were available, making it difficult to ascertain whether some of these disorders were caused by conditions that predated the oil spill. Nevertheless, the relationships between reported levels of oil spill exposure and both depression and mental distress were consistent with estimates reported in other disaster studies. A second important limitation is that these data are cross-sectional, and the results should be interpreted with appropriate caution. An alternative explanation of the study’s results is that individuals with higher levels of mental health problems tended to report more exposure to the oil spill. Third, there is no standard method to measure exposure to an oil spill. In the absence of biomarkers or other objective measures, studies often rely on survey data or geographic proximity to the oil spill as proxy measures of exposure. Thus, there is a wide variety of approaches used to characterize exposure across studies, making comparisons difficult. We chose a method that combined several questionnaire items into two composite variables, which essentially assessed the degree of impact of exposure on subjects’ lives. Nevertheless, this study employed many of the same items used in other oil spill studies, and the results appear to be robust regardless of the method used. Fourth, this study included only women and had a response rate of 45%; therefore, comparisons with populations of both sexes and generalization to the population at large should be made with caution. Fifth, it is possible that mental health outcomes were underreported because of stigmatization or lack of participation by those most heavily affected, which would make the present estimates conservative. However, selection bias whereby potential respondents were more inclined to participate because they had health-related complaints may have offset the underreporting. Finally, the study accepted volunteers as well as randomly sampled participants, potentially introducing selection bias into the results. However, volunteers comprised < 5% of the sample. Excluding the volunteers (data not shown) produced no meaningful changes in the results.

## Conclusion

Despite the abovementioned limitations, the WaTCH Study is a large, population-based study of women in a particularly vulnerable area of the country that *a*) has improved on estimates of survey-derived exposures to oil spills by capturing two distinct aspects of exposure: physical and economic exposure, and *b*) has provided important information on the mental health correlates of the *Deepwater Horizon* oil spill that could lead to improved planning and disaster mitigation in the future.
